# Pressure-tuning the quantum spin Hamiltonian of the triangular lattice antiferromagnet Cs_2_CuCl_4_

**DOI:** 10.1038/s41467-019-09071-7

**Published:** 2019-03-06

**Authors:** S. A. Zvyagin, D. Graf, T. Sakurai, S. Kimura, H. Nojiri, J. Wosnitza, H. Ohta, T. Ono, H. Tanaka

**Affiliations:** 1 Dresden High Magnetic Field Laboratory (HLD-EMFL), Helmholtz-Zentrum Dresden-Rossendorf, 01328 Dresden, Germany; 2 National High Magnetic Field Laboratory, Florida State University, Tallahassee, FL 32310 USA; 3 Research Facility Center for Science and Technology, Kobe University, Kobe, 657-8501 Japan; 4 Institute for Materials Research, Tohoku University, Sendai, 980-8578 Japan; 5 Institut für Festkörper- und Materialphysik, TU Dresden, 01062 Dresden, Germany; 6 Molecular Photoscience Research Center, Kobe University, Kobe, 657-8501 Japan; 7 Department of Physical Science, Osaka Prefecture University, Osaka, 599-8531 Japan; 8 Department of Physics, Tokyo Institute of Technology, Tokyo, 152-8551 Japan

## Abstract

Quantum triangular-lattice antiferromagnets are important prototype systems to investigate numerous phenomena of the geometrical frustration in condensed matter. Apart from highly unusual magnetic properties, they possess a rich phase diagram (ranging from an unfrustrated square lattice to a quantum spin liquid), yet to be confirmed experimentally. One major obstacle in this area of research is the lack of materials with appropriate (ideally tuned) magnetic parameters. Using Cs_2_CuCl_4_ as a model system, we demonstrate an alternative approach, where, instead of the chemical composition, the spin Hamiltonian is altered by hydrostatic pressure. The approach combines high-pressure electron spin resonance and r.f. susceptibility measurements, allowing us not only to quasi-continuously tune the exchange parameters, but also to accurately monitor them. Our experiments indicate a substantial increase of the exchange coupling ratio from 0.3 to 0.42 at a pressure of 1.8 GPa, revealing a number of emergent field-induced phases.

## Abstract (web summary)

Theoretical studies of quantum magnetism typically assume idealised lattices with freely tunable parameters, which are difficult to realise experimentally. Zvyagin et al. perform challenging measurements at high pressures to tune and to accurately monitor the exchange parameters of a triangular lattice antiferromagnet.

## Introduction

The interplay between geometrical frustration, quantum fluctuations, and magnetic order is one of the central issues in condensed matter physics. In 1973, developing the resonating valence bond (RVB) theory, Anderson proposed that quantum fluctuations in magnetic structures on an isotropic triangular lattice can be sufficiently strong to destroy the magnetic order, resulting in a two-dimensional (2D) fluid of mobile spin pairs correlated together into singlets^1^. This state was introduced as a RVB quantum spin liquid, contrary to the valence-bond solid (VBS), with the ground state condensed into a spin lattice. The Anderson’s hypothesis has triggered a cascade of extensive theoretical and experimental studies, resulting in the discovery of numerous exotic quantum states and highly unusual field-induced phenomena^2^.

**Fig. 1:**
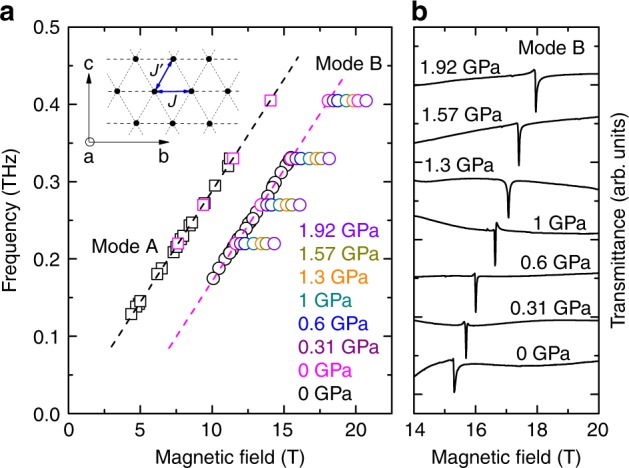
Pressure dependence of ESR excitations in Cs_2_CuCl_4_ (*T* = 1.9 K, *H*||*b*). **a** Frequency-field diagrams of ESR excitations at different pressures. The data denoted in black are taken from ref. ^6^ (*T* = 1.5 K; 0 GPa). Dashed lines correspond to the fit results (see text for details). Source data are provided as a Source Data file. The inset shows a schematic picture of magnetic sites and exchange couplings in a triangular layer of Cs_2_CuCl_4_. **b** ESR spectra (mode B) taken at 330 GHz at different pressures (the spectra are offset for clarity)

The spin-1/2 triangular-lattice Heisenberg antiferromagnet (AF) represents one of the most important groups of the family of low-D quantum frustrated magnets. For the general case of spatially anisotropic triangular AF, the spin Hamiltonian is given as

1 $${\cal H} = J\mathop {\sum}\limits_{\langle i,j\rangle } {\mathbf{S}}_i \cdot {\mathbf{S}}_j + J^{\prime}\mathop {\sum}\limits_{\langle i,j^{\prime}\rangle } {\mathbf{S}}_i \cdot {\mathbf{S}}_{j^{\prime}},$$

where **S**_*i*_, **S**_*j*_, and **S**_*j*′_ are spin-1/2 operators at sites *i*, *j*, and *j*′, and *J* and *J*′ are the exchange interactions on the horizontal and diagonal bonds, respectively (Fig. 1a, inset). In spite of this simple model, such systems are shown to possess a very rich and not fully understood phase diagram, which can be interpolated between decoupled spin-chain (*J*′ = 0), isotropic triangular (*J*′/*J* = 1), and unfrustrated square (*J* = 0) lattices. It is expected that transitions from one state to another occur in between these well-defined cases, but many details of this evolution (e.g., critical coupling ratios) still remain a matter of debate^3,4^. The magnetic phase diagram predicts a variety of exotic phases, with the 1/3 saturation-magnetization plateau as the most exciting magnetic property^5^.

The largest hindrance to experimentally check theoretical predictions on the unusual magnetic properties of spin-1/2 triangular lattice Heisenberg AFs is the very limited number of materials with appropriate (ideally tuned) sets of parameters, currently available for measurements. In spite of the recent progress in synthesizing spin-1/2 triangular-lattice materials (see e.g., ref. ^2^ and references therein), the two compounds, Cs_2_CuCl_4_ and Cs_2_CuBr_4_ (with *J*′/*J* ≃ 0.30 and 0.41, respectively^6^), remain among the most prominent representatives of this family of frustrated materials. One obvious approach to tune the spin Hamiltonian of these systems is to vary their chemical composition^7,8^. However, experiments on the solid solution Cs_2_CuCl_4−*x*_Br_*x*_ (with Br content ranging from 0 to 4) revealed a pronounced difference in the Cu coordination when increasing *x*, resulting in a discontinuous evolution of its crystal structure^9^.

The high-pressure technique is known as a powerful means to modify magnetic properties and parameters of exchange coupled spin systems (see e.g., refs. ^10–19^). On the other hand, another important task is to precisely measure these parameters. This becomes particularly challenging for low-D spin systems, whose spin Hamiltonian is strongly affected by quantum fluctuations. One solution to solve this problem is to suppress quantum fluctuations by strong-enough magnetic fields, and then to use the harmonic spin-wave theory for a description of the excitation spectrum^6,20^.

Here, we combine high-pressure high-field electron spin resonance (ESR) and radio frequency (r.f.) susceptibility measurements, allowing us not only to quasi-continuously change the exchange parameters *J* and *J*′, but also to accurately monitor them. We use Cs_2_CuCl_4_ as a model system. We show that the application of pressure increases significantly the exchange coupling parameters in this compound, triggering, at the same time, the emergence of field-induced low-temperature magnetic phases, absent at zero pressure.

## Results

### High-pressure ESR measurements

**Fig. 2:**
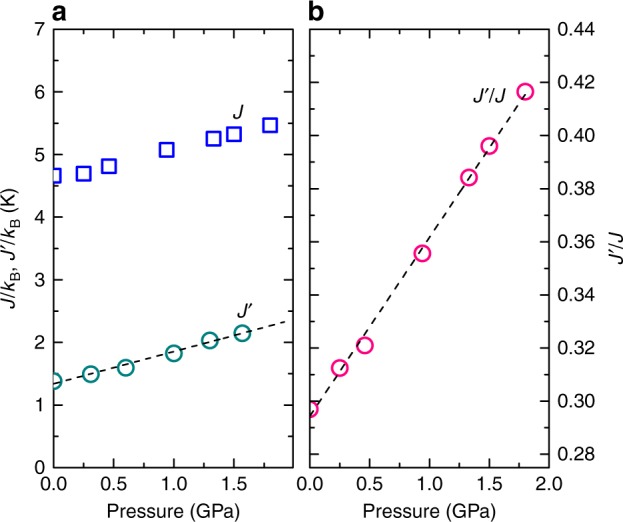
Pressure-driven tuning of the spin-Hamiltonian parameters in Cs_2_CuCl_4_. **a** Pressure dependence of the exchange coupling parameters *J*′ and *J* (circles and boxes, respectively). The dashed line corresponds to a linear fit to the *J*′ data (see text for details).  **b** Pressure dependence of the exchange coupling ratio  *J*′/*J*. The dashed line corresponds to a linear fit to the *J*′/*J* data (see text for details). Source data are provided as a Source Data file

To determine the dependence of the coupling parameters of Cs_2_CuCl_4_ on the applied pressure, we used the procedure employed in ref. ^6^, when the excitation spectrum is measured above the saturation field *H*_sat_. In the case of the staggered Dzyaloshinskii–Moriya (DM) interaction, the ESR spectrum should consist of two modes, which correspond to magnetic excitations at the center and at the boundary of the unfolded Brillouin zone (a.k.a. the relativistic and exchange modes, respectively). Such modes were previously observed in Cs_2_CuCl_4_^6^ (black symbols in Fig. 1a). The field dependence of the relativistic mode A for $H \,\gtrsim \, J/g\mu _{\mathrm{B}}$ can be described using the equation ℏ*ω*_A_ = *gμ*_B_*H*, where ℏ is the reduced Planck constant, *ω* is the excitation frequency, *μ*_B_ is the Bohr magneton, and *g* = 2.06 is the *g* factor (the fit results are shown in Fig. 1a by the black dashed line). On the other hand, the frequency-field diagram of mode B can be described using the equation ℏ*ω*_B_ = *gμ*_B_*H* − Δ_B_ (magenta dashed line in Fig. 1a) with the same *g* factor as for the mode A. Most importantly, the difference between the excitation energies for modes A and B (Δ*ω*_AB_ ≡ Δ_B_) is determined by *J*′: *J*′ = ℏΔ*ω*_AB_/4, allowing us to measure *J*′ directly. The experiment revealed a shift of the mode B towards higher field when the pressure is applied. The pressure dependence of *J*′ is shown in Fig. 2a, evident in a significant, almost 70%, increase of *J*′ at 1.92 GPa.

### High-pressure TDO measurements

**Fig. 3:**
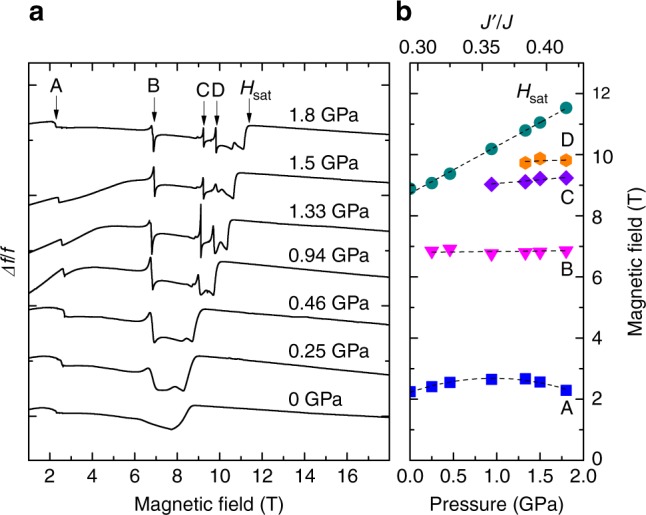
Pressure evolution of magnetic properties of Cs_2_CuCl_4_ obtained by means of TDO technique (*T* = 350 mK, *H*||*b*). **a** Pressure dependence of the TDO frequency changes Δ*f*/*f* in response to the magnetic field (the data are offset for clarity). **b** Dependencies of TDO frequency anomalies on the applied pressure. The calculated exchange coupling ratio *J*′/*J* is shown on the top scale (see text for details). Lines are guides for the eye. Source data are provided as a Source Data file

Knowing *J*′ and the saturation field *H*_sat_, we can determine *J*, using the expression *gμ*_B_*H*_sat_ = 2*J*(1 + *J*′/2*J*)^2^. To measure the saturation field of Cs_2_CuCl_4_, we employ a tunnel-diode-oscillator (TDO) technique (see Methods). The variations of the TDO circuit resonant frequency Δ*f*/*f* (which is proportional to the magnetic susceptibility) as a function of the magnetic field applied along the *b* axis at different pressures are shown in Fig. 3a. In strong magnetic fields, the TDO frequency is almost constant, indicating the transition of Cs_2_CuCl_4_ into the fully spin-polarized phase with saturated magnetization^21^. The experiment revealed that with increasing pressure the saturation field moves toward higher magnetic fields. The dependence of *H*_sat_ on the applied pressure is shown in Fig. 3b.

Based on the combined ESR and TDO data, for zero pressure we obtained *J*′/*k*_B_ = 1.38 K and *J*/*k*_B_ = 4.66 K (*J*′/*J* ≃ 0.3), which perfectly agrees with the previous estimates^6^. Results of a linear fit to the *J*′ dependence (dashed line in Fig. 2a) were used to calculate *J* at different pressures. *J*′, *J*, and *J*′/*J* as functions of the applied pressure are shown in Fig. 2. The *J*′/*J* dependence can be described using the empirical equation *J*′/*J* = 0.294(2) + 0.067(2)⋅*P* (dashed line in Fig. 2b), where *P* is the applied pressure (GPa). For 1.8 GPa, we obtained *J*′/*k*_B_ = 2.28 K, *J*/*k*_B_ = 5.47 K, and *J*′/*J* ≃ 0.42, indicating a remarkable, by 40%, increase of the *J*′/*J* ratio. Based on this fit, the application of a pressure of 3.6 GPa (where Cs_2_CuCl_4_ undergoes a structural phase transition^22^) would allow one to reach $J^{\prime}/J \simeq 0.53$ (which corresponds to approximately 180% of the zero-pressure value).

## Discussion

Apart from the shift of the saturation field, our experiment revealed a number of magnetic anomalies, which are absent in Cs_2_CuCl_4_ at zero pressure (Fig. 3a). The observed magnetic anomalies can be caused by changes in the dynamics of critical fluctuations in the vicinity of field-induced phase transitions^23^, resulting in changes of real and imaginary components of the magnetic susceptibility. Although no signature of the 1/3 magnetization plateau was revealed, our observation (Fig. 3b) resembles the cascade of field-induced phase transitions in quasi-2D Cs_2_CuBr_4_^24^, evident of a complex picture of magnetic interactions, including different perturbation terms (a remarkable sensitivity of the magnetic phase diagrams of Cs_2_CuCl_4_ to the direction of the applied magnetic field^21^ strongly suggests an important role not only spatial (*J*′ ≠ *J*), but also spin-space (asymmetric DM interaction) components of the magnetic anisotropy; the latter appear to be of the same order of magnitude as the interplane exchange interaction *J*″^20^, inducing strongly relevant perturbations^25^).

For the magnetic field applied along the *b* axis, the zero-pressure magnetic phase diagram contains four low-temperature phases^21^. At small field below *T*_*N*_ = 0.62 K, the system is in the incommensurate phase with a spiral ground state^26^ dominantly determined by the DM anisotropy (“DM spiral”)^25^. In this phase, the spins are located almost in the *b*–*c* plane with the spiral propagating along the *b* axis^26^. Remarkably, at about 2.3 T the effect of the DM interaction becomes irrelevant and the system undergoes a transition into the commensurate coplanar AF phase with spins more correlated in *a*–*b* planes (the corresponding correlations are determined by *J*″ and *J*)^25^. These two magnetic phases are stabilized by quantum fluctuations. The commensurate coplanar AF state is realized in a relatively wide field range, followed by two successive high-field transitions: into the noncoplanar cone phase and then, with further increase of the applied magnetic field, into the fully spin-polarized magnetically saturated phase (both phases are favored classically).

**Fig. 4:**
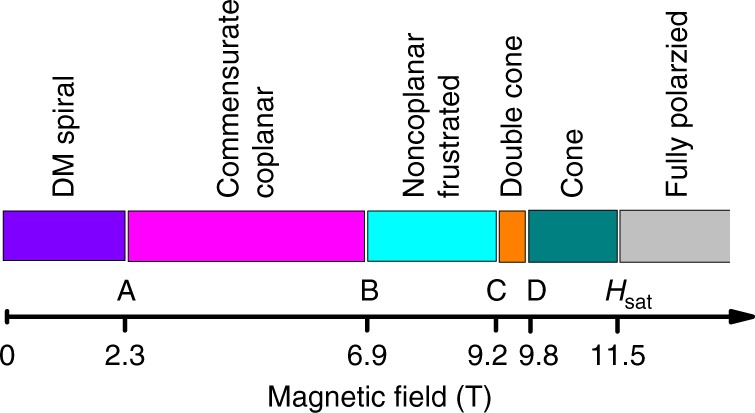
The proposed phase diagram of Cs_2_CuCl_4_ under pressure 1.8 GPa (which corresponds to *J*′/*J* = 0.42)

What happens when pressure is applied? Apart from the shift of the saturation field, our experiment revealed a number of magnetic transitions, absent at zero pressure (Fig. 3a). The proposed magnetic phase diagram for 1.8 GPa is shown in Fig. 4. Similar to that at zero pressure, at low field the system is in the DM spiral phase. The DM spiral phase is suppressed by the magnetic field at about 2.2–2.6 T (the anomaly A in Fig. 3a corresponds to this transition), resulting in the commensurate coplanar AF phase with spins predominantly correlated in the *a*–*b* plane. Applied pressure makes the *J*′ term more and more relevant, tending to suppress the coplanar nature of magnetic correlations. As a combined effect of the applied magnetic field (partially suppressing quantum order) and pressure (enhancing the interplane correlations), at about 6.9 T the system undergoes a transition into a noncoplanar (presumably) frustrated phase. The observed anomaly B corresponds to this transition.

For a spatially anisotropic triangular lattice AF in magnetic fields near the saturation, theory^27^ predicts a particular rich phase diagram, with ground states ranging from an incommensurate noncoplanar chiral cone to a commensurate coplanar *V* state. The transformation between these two states involves two intermediate phases. One of them is a coplanar incommensurate order, while another one is a noncoplanar double-**Q** spiral order (double-cone state). The latter is characterized by the broken *Z*_2_ symmetry between two magnon condensates at ±*Q* (where *Q* is the ordering wave vector) and can coexist with the single-cone phase in a relatively narrow range of *J*′/*J*, but at smaller fields. In Cs_2_CuCl_4_ at zero pressure, the transition into the single-cone phase was revealed between 8 and 9 T below 300 mK^21^. Due to the increase of exchange coupling parameters, the applied pressure shifts the upper boundary of the temperature-field phase diagram to higher temperatures. Because of that, the transition into the single-cone phase can be observed at higher temperatures. Based on this assumption, the anomalies C and D (Fig. 3). can be interpreted as transitions into the double- and single-cone phases, respectively (Fig. 4). A tiny feature immediately before saturation might indicate the involvement of other higher-order perturbation factors (e.g., next-nearest-neighbor interactions^28^ or the interplane frustration mentioned above^29^).

Our observations call for systematic high-pressure magneto-structural (such as nuclear magnetic resonance and neutron diffraction) studies of Cs_2_CuCl_4_, which would allow one to verify the proposed phase diagram. Apart from exact identification of the nature of the observed high-pressure phases, another important task would be the search for the field-induced 1/3 magnetization plateau, which can be expected with further increase of *J*′/*J* moving the system towards the isotropic (*J*′/*J* = 1) limit^27^. It would be also very interesting to measure the pressure-driven evolution of the spin Hamiltonian in the isostructural compound Cs_2_CuBr_4_ and to compare the results with that in Cs_2_CuCl_4_.

To conclude, we demonstrated an effective strategy to control the spin Hamiltonian of a spin-1/2 antiferromagnet on a triangular lattice with hydrostatic pressure. With increasing pressure, for Cs_2_CuCl_4_ our experiments revealed a substantial increase of the exchange coupling parameters, accompanied by the emergence of (at least) two field-induced phases. These phases can be tentatively interpreted as noncoplanar frustrated and double-cone states, merging the low-field commensurate coplanar and high-field single-cone phases revealed previously. Our approach provides robust means for investigating the complex interplay between geometrical frustration, quantum fluctuations, and magnetic order (especially, close to quantum phase transitions), paving the way towards controlled manipulation of the spin Hamiltonian and magnetic properties of frustrated spin systems.

## Methods

### Single-crystal growth

Single-crystal samples of Cs_2_CuCl_4_ were grown by the slow evaporation of an aqueous solution of CsCl and CuCl_2_ in the mole ratio 2:1.

### High-pressure TDO

High-pressure TDO measurements were conducted at the National High Magnetic Field Laboratory (Florida State University) in magnetic fields up to 18 T using a TDO susceptometer^14,15,30^ tuned to operate at a resonant frequency of 51 MHz. Magnetic field was applied along the *b* axis of the crystal. A sample with a length of ~1.5 mm was placed in a copper-wire coil with diameter ~0.8 mm and height ~1 mm. The coil and sample were surrounded with Daphne 7575 oil (Idemitsu Kosan Co., Ltd.) and encapsulated in a Teflon cup which was inserted into the bore of a piston-cylinder pressure cell constructed from a chromium alloy (MP35N). The coil acts as an inductor in a diode-biased self-resonant LC tank circuit. During the field sweep, changes in the sample magnetic permeability lead to changes in the inductance of the oscillator tank coil, and, hence, to changes in the TDO circuit resonant frequency Δ*f*. The frequency changes were detected as a function of the magnetic field at different pressures. The pressure created in the cell was calibrated at room temperature and again at low temperature using the fluorescence of the R1 peak of a small ruby chip as a pressure marker^31^ with accuracy better than ±0.015 GPa. The pressure cell was immersed directly into ^3^He, allowing TDO measurements down to 350 mK. Temperature was measured using a calibrated Cernox thermometer. Transition fields were measured with accuracy better than ±0.5%.

### High-pressure ESR

High-pressure ESR measurements of Cs_2_CuCl_4_ were performed at the High Field Laboratory for Superconducting Materials, Institute for Material Research (IMR), Tohoku University using a transmission-type ESR probe^32,33^ with oversized waveguides and a 25 T cryogen-free superconducting magnet^34,35^. Gunn-oscillators, operated at frequencies 220, 270, 330, and 405 GHz, were employed as radiation sources. A hot-electron InSb bolometer cooled down to 4.2 K was used as a detector. Magnetic field was applied along the *b* axis of the crystal. Experiments were performed at a temperature of 1.9(1) K; the temperature was measured using a calibrated Cernox thermometer. A cylinder-shaped crystal with approximate dimensions of 9 mm in length by 5 mm in diameter was immersed in a Teflon cup filled with Daphne 7474 oil (Idemitsu Kosan Co., Ltd.) as pressure medium. A two-section piston cylinder pressure cell made from NiCrAl (inner cylinder) and CuBe (outer sleeve) has been used. The key feature of the pressure cell is the inner pistons, made of ZrO_2_ ceramics; this material has low loss for electromagnetic radiation with frequency up to 800 GHz. The change of the superconducting transition temperature of tin was used to calibrate the applied pressure^36^; the transition temperature was detected by AC magnetic susceptibility measurements. Applied pressure was calculated using the relation between the load at room temperature and the pressure obtained at around 3 K^32^; the pressure calibration accuracy is better than ±0.05 GPa. ESR line position (mode B) was measured with accuracy better than ±0.2%. In our experiments, we assume that the accuracies estimating *J*′, *J*, and *J*′/*J* including all possible error sources, are better than ±1%, ±4%, and ±5%, respectively.

## Data Availability

The data that support the findings of this study are available from the corresponding author upon reasonable request. The source data underlying Figs. 1a, 2a, b, and 3b are provided as Source Data files.

## Supplementary data

Source Data
